# Unveiling the untreated: development of a database algorithm to identify potential Fabry disease patients in Germany

**DOI:** 10.1186/s13023-024-03258-y

**Published:** 2024-07-09

**Authors:** Max J. Hilz, Nicole Lyn, Felix Marczykowski, Barbara Werner, Marc Pignot, Elvira Ponce, Joseph Bender, Michael Edigkaufer, Pronabesh DasMahapatra

**Affiliations:** 1 University of Erlangen-Nuremberg, Erlangen, Germany; 2 Icahn School of Medicine at Mount Sinai, New York, NY, USA; 3 Sanofi, Cambridge, MA USA; 4 Oracle Life Sciences, Munich, Germany; 5 Team Gesundheit GmbH, Essen, Germany; 6 ZEG Berlin – Center for Epidemiology and Health Research, Berlin, Germany; 7 Sanofi, Frankfurt, Germany

**Keywords:** Algorithm, BKK German Sickness Fund Database, Early diagnosis, Fabry disease, Logistic regression modelling

## Abstract

**Background:**

Fabry disease (FD), an X-linked lysosomal storage disorder, is caused by mutations in the gene encoding α-galactosidase A, resulting in lysosomal accumulation of globotriaosylceramide and other glycosphingolipids. Early detection of FD is challenging, accounting for delayed diagnosis and treatment initiation. This study aimed to develop an algorithm using a logistic regression model to facilitate early identification of patients based on ICD-10-GM coding using a German Sickness Fund Database.

**Methods:**

The logistic regression model was fitted on a binary outcome variable based on either a treated FD cohort or a control cohort (without FD). Comorbidities specific to the involved organs were used as covariates to identify potential FD patients with ICD-10-GM E75.2 diagnosis but without any FD-specific medication. Specificity and sensitivity of the model were optimized to determine a likely threshold. The cut-point with the largest values for the Youden index and concordance probability method and the lowest value for closest to (0,1) was identified as 0.08 for each respective value. The sensitivity and specificity for this cut-point were 80.4% and 79.8%, respectively. Additionally, a sensitivity analysis of the potential FD patients with at least two codes of E75.2 diagnoses was performed.

**Results:**

A total of 284 patients were identified in the potential FD cohort using the logistic regression model. Most potential FD patients were < 30 years old and female. The identification and incidence rates of FD in the potential FD cohort were markedly higher than those of the treated FD cohort.

**Conclusions:**

This model serves as a tool to identify potential FD patients using German insurance claims data.

**Supplementary Information:**

The online version contains supplementary material available at 10.1186/s13023-024-03258-y.

## Introduction

Fabry disease (FD) is an X-linked lysosomal storage disorder of glycosphingolipid metabolism caused by rare inborn mutations in the *GLA* gene encoding α-galactosidase A, which causes progressive accumulation of globotriaosylceramide (GL-3 or Gb3) and its deacylated forms, globotriaosylsphingosine (lyso-GL-3 or lyso-Gb3) substrates in lysosomes [1, 2]. This can result in cell damage leading to irreversible progressive damage to major organ systems, including the vascular system, central and peripheral nervous systems, skin, intestine, heart, and kidneys [3, 4]. Patients with FD experience various symptoms, including neuropathic pain, heat intolerance, gastrointestinal discomfort, decreased sweating, angiokeratoma, and fatigue [3]. The estimated FD prevalence ranges between 1 in 40,000–117,000 live births globally [5] and 1–5 per 10,000 persons in Germany [6]. However, the reported numbers might be underestimated [3, 4] because underdiagnosis of female patients and patients with atypical disease manifestations may contribute to underreporting of the actual prevalence.

The natural history of FD shows heterogeneity in disease presentation across its two phenotypes: classic (early-onset) and non-classic (late-onset) [3, 7]. Clinical variability of different mutations, variability in disease severity, intersex variability, and symptom-onset time contribute to diagnostic and prognostic challenges [8, 9]. Early onset of FD symptoms should facilitate prompt diagnosis. However, recognizing early manifestations in clinical practice is challenging, and definitive diagnosis may be delayed by ~15 years; patients with late-onset FD exhibit symptoms mostly in adulthood [3, 10]. Early treatment initiation has greater potential for clinical benefits. Enzyme replacement therapy at an early age may slow or prevent irreversible changes in renal and cardiac systems [11–13]. Oral chaperone therapy is available for a subset of patients with amenable *GLA* variants [14].

The proportion of patients who are likely to have FD but not diagnosed/treated is relatively high [15]. In Germany, there is no unique International Statistical Classification of Diseases and Related Health Problems (ICD) code specific to FD. To facilitate early diagnosis and treatment of patients with FD, this study aimed to develop a patient-identification algorithm using logistic regression modelling to identify patients who are likely to have FD and are not treated with FD-specific medication (potential FD patients) using the BKK (*BetriebsKrankenKassen*, company health insurance funds) German Sickness Fund Database.

## Methods

### Study design

This retrospective analysis was conducted using the BKK German Sickness Fund Database, which is representative of 5.4 million insured individuals registered in 2010–2017. Membership in the statutory health insurance scheme is compulsory for 87% of the German population [16]. While the primary purpose of these data is for reimbursement, they can also serve as a source for research. The available data included patient demographics, outpatient/ambulatory treatment information, including the ICD, 10^th^ revision (ICD-10) diagnosis code, inpatient treatment/hospitalization data, prescription information, and details of sickness benefits. All patient-level data were anonymized to comply with German data protection regulations. Eligible patients were followed up from the beginning of enrollment (Jan 01, 2010) until loss owing to end of continuous enrollment (based on available patient records/data from the database), death, or end of the observation period (Dec 31, 2017).

### Study population

Three cohorts were identified in the BKK German Sickness Fund Database: [A] Patients with FD having FD-specific prescriptions (treated FD cohort), [B] patients with potential FD without FD-specific prescriptions (potential FD cohort), and [C] a sample of insured subjects without FD (control cohort).

### Identification of treated FD cohort, potential FD cohort, and control cohort

**Fig. 1:**
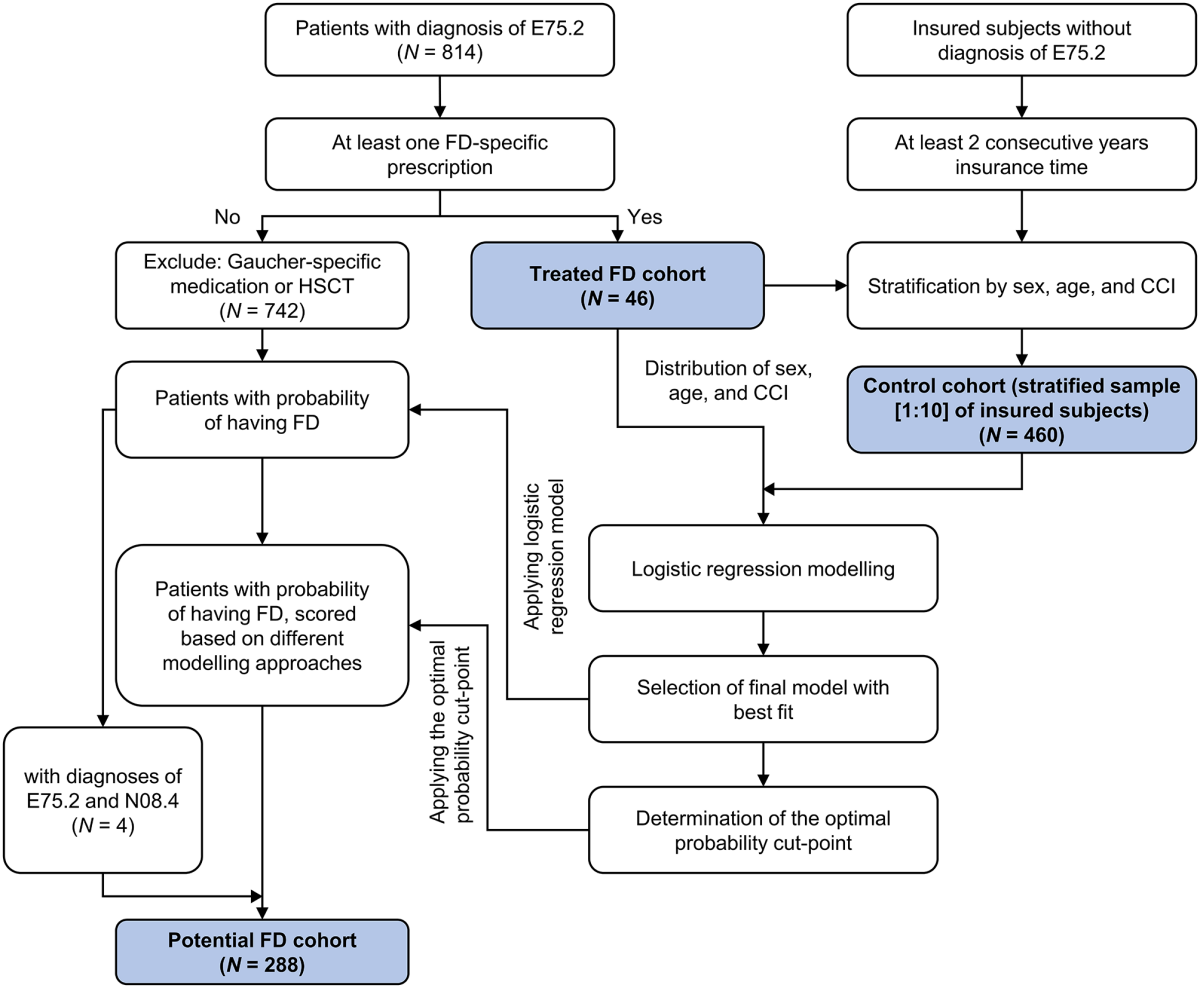
Identification of treated FD cohort, potential FD cohort, and control cohort. *Total patients with Gaucher or HSCT (*n* = 26) were excluded. Hence, 742 patients were further evaluated. E75.2 represents sphingolipidoses, including Fabry [–Anderson], Gaucher disease, Krabbe, Niemann–Pick, Farber syndrome, Metachromatic leukodystrophy, and Sulfatase deficiency; N08.4 represents glomerular disorders in other endocrine, nutritional, and metabolic diseases, including glomerular disorders associated with amyloidosis, FD, or lecithin-cholesterol acyltransferase deficiency. CCI, Charlson Comorbidity Index; FD, Fabry disease; HSCT, hematopoietic stem cell transplant

[A] Treated FD cohort: All patients with a diagnosis of ICD-10, German Modification (ICD-10-GM) E75.2 (other sphingolipidoses, including Fabry [–Anderson] disease, Gaucher disease, Krabbe disease, Niemann–Pick disease, Farber syndrome, Metachromatic leukodystrophy, and Sulfatase deficiency) were identified (Fig. 1). Only confirmed diagnoses (ICD-10-GM codes, marked by “G” [confirmed] or “Z” [asymptomatic condition after diagnosis]) were considered in the data on outpatient care visits. For hospital visits, the main and secondary diagnoses were checked. Patients had either one diagnosis in hospital data (main/secondary diagnosis) or one confirmed outpatient diagnosis claim. Because the ICD-10-GM E75.2 code is not unique for FD but also includes other lysosomal storage diseases, an additional inclusion criterion of at least one prescription of a FD-specific medication (Anatomical Therapeutic Chemical code: agalsidase alfa [A16AB03], agalsidase beta [A16AB04], and migalastat [A16AX14]) during the identification period was applied to identify treated FD patients. Individuals with at least one Gaucher-specific medication or having undergone one hematopoietic stem cell transplant were classified as not having FD and were excluded.

[B] Potential FD cohort: Potential patients with ICD-10-GM E75.2 diagnosis but without any FD-specific medication during the observational period were identified using a logistic regression model (Fig. 1).
[C] Control cohort: Patients with ICD-10-GM E75.2 diagnosis during the study period were excluded from building this study cohort. Furthermore, insured subjects without an insurance period of at least 2 consecutive years were excluded. A random sample (1:10) stratified by age, sex, and Charlson Comorbidity Index (CCI) [17] was identified (Fig. 1). The age, sex, and CCI distributions were taken from the treated FD cohort [A].

### Development of a logistic regression model

The probability of having FD was predicted using a logistic regression model with the disease being an outcome variable and defined covariates being explanatory variables (Table 1). The logistic regression model was fitted on a binary outcome variable based on either treated FD patients or control subjects (non-FD patients). The advantage of using these cohorts for model development was that the disease status (FD diagnosis) of the participants was known; this can help evaluate the sensitivity and specificity of the model.

**Table 1:** Covariates assessed for the model development to identify the potential FD cohort

Covariates	ICD-10-GM Code	Explanation
Age at index	-	Age at the first E75.2 diagnosis during the observational period (treated FD patients) Age 1 year after beginning of the insurance time (control cohort)
Sex	-	Male, female
Follow-up time		Follow-up time from the index date until the end of observation time/end of study
Other FD typical comorbidities	I73.8 M79.2 F45.41 R52.1 R52.2 R20.- L74.4 H90.- H91.- H93.1 H81.0-9 H82.- R42.-	Other specified peripheral vascular diseases Neuralgia and neuritis, unspecified Pain disorder exclusively related to psychological factors Chronic uncontrollable pain Other chronic pain Disturbances of skin sensation Anhidrosis Conductive and sensorineural hearing loss Other and unspecified hearing loss Tinnitus Disorders of vestibular function Vertiginous syndromes in diseases classified elsewhere Dizziness and giddiness
Dermatological involvement*	*D23*	*Other benign neoplasm of the skin*
Gastrointestinal involvement	A09.- K52.9	Infectious gastroenteritis and colitis, unspecified Noninfective gastroenteritis and colitis, unspecified
Ophthalmological involvement	H18.5 H35.0 H26.- H28.- Q12.0	Hereditary corneal dystrophies Other retinal disorders Other cataract Cataract in diseases classified elsewhere Congenital lens malformations
Renal involvement	R80.- N18.- N17.- N19.-	Proteinuria Chronic kidney disease Acute kidney failure Unspecified kidney failure
Cardiac involvement	I42.- I51.7 I11.0- I50.- R57.0 R00.1 I48.- R00.0 I47 I49.9	Cardiomyopathy Cardiomegaly Hypertensive heart disease with (congestive) heart failure Heart failure Cardiogenic shock Bradycardia, unspecified Atrial fibrillation and atrial flutter Tachycardia, unspecified Paroxysmal tachycardia Cardiac arrhythmia, unspecified
Vascular involvement*	*I70.0*	*Atherosclerosis of the aorta*
Cerebrovascular involvement	G45.- I63.- I80.- I64.-	Cerebral transitory ischemia and related syndromes Stroke Thrombosis, phlebitis, and thrombophlebitis Stroke, not referred to as bleeding or infarction
Neuropsychiatric involvement*	*F06.3* *F25.-* *F32.-* *F33.-* *F34.-* *F41.2* *F41.-*	*Organic mood disorders* *Schizoaffective disorders* *Depressive episode* *Recurrent depressive disorder* *Persistent mood disorders* *Anxiety and depressive disorder, mixed* *Other anxiety disorders*

*The italicized covariates were not selected in the final model

FD, Fabry disease; ICD-10-GM, International Statistical Classification of Diseases and Related Health Problems, 10^th^ revision, German Modification

Potential covariables for the model included comorbidities typical for FD, identified based on the Ortiz “Management and treatment guidelines,” and common comorbidities were assigned to a high-level organ involvement category (Table 1) [18]. A binary variable for each organ involvement was generated and set to “yes” (1) in case comorbidity of the organ involvement category was identified during the total follow-up period of the patient. ICD-10 codes were identified for these comorbidities (Table 1), and the comorbidity occurrences in the treated FD cohort were noted. These comorbidities were categorized as  “very important,” “important,” and “less important” based on the review of FD specificity by a clinician.

Based on the importance of these comorbidities, two approaches were considered for evaluating covariates for the model: approach one considered comorbidities for each organ involvement rated as “very important” or “important” according to qualitative review by a clinician; the second approach additionally included “less important” variables that were non-specific to FD, such as abdominal/pelvic pain (Table 1). Some ICD-10 codes (comorbidities) were excluded from both models, as they were not considered appropriate or had small counts. In addition, variables for sex, age at index, and follow-up time (quarters) were included in both models (Table 1).

Logistic regression was carried out using the SAS software (version 9.4). A stepwise logistic regression method was employed for model selection, with significance levels of 0.30 and 0.35 for variable inclusion and variable exclusion, respectively [19]. The model with the smallest value for the Akaike information criterion (AIC) and the largest value for area under the receiver operating characteristic (ROC) curve was selected. After the best fit model was identified, an optimal cut-point was determined for the probability of having FD, as estimated by the model. A treated FD patient with the probability of having FD higher than the identified cut-point was confirmed as a true positive. A patient from the control cohort was confirmed as a true negative if the probability was lower than the cut-point. For different cut-points, the rates of true positive (sensitivity) and true negative (specificity) cases were calculated, and the following parameters were maximized: Youden-Index, closest to (0,1), and the concordance probability method [19, 20].

The logistic regression model was used to generate the probability of having FD for each potential patient. Only patients whose estimated probability of having FD was higher than the cut-point were assumed to have FD.

Additionally, the patients with E75.2 diagnoses and “Glomerular disorders in other endocrine, nutritional, and metabolic diseases” (ICD-GM-10 N08.4) were included in the potential FD cohort irrespective of the estimated probability of having FD, with the assumption that they had FD.

### Study period

The study duration (Jan 01, 2010, to Dec 31, 2017) included two identified time periods: pre-index and post-index. Information on drug prescriptions and hospital visits were available on daily basis. In contrast, diagnoses made by practitioners were available quarterly. For the treated and potential FD cohorts, the index date was defined as the date of the first visible E75.2 diagnosis during the study period, and the quarter of the first E75.2 diagnosis was documented as index quarter (Supplemental Fig. 1). For the control cohort, the index date was defined as the date 1 year after the start of the insurance period. The time before index (pre-index) comprised four quarters before the index quarter and might include the year 2009, depending on the first visible diagnosis. The time after index (post-index) included the index quarter and the following three quarters. Insured subjects with an observation period of at least four quarters before and after the index date were deemed eligible for inclusion in the control cohort. Given the small sample size of the treated and potential FD patients, the pre-index and post-index follow-up periods were not considered part of the inclusion criteria.

### Statistical analysis

Categorical variables are presented as *n* (%). Continuous variables are presented as mean (standard deviation [SD]). Furthermore, the minimum, 25^th^ percentile, median, 75^th^ percentile, and maximum values are also reported. All treated FD patients were considered as prevalent. However, a patient without E75.2 diagnosis in the entire pre-index period (1 year before their index date) during the study period was considered as an incident. Incidence and prevalence rates of FD for treated patients were estimated. Similarly, incidence and identification rates of potential patients (identified by the model) were separately assessed. The rates were standardized by age and sex as per those of the German statutory health insured population using data from the German Ministry of Health [21]. Incidence and prevalence/identification rates are reported per 100,000 persons and were estimated for the overall and each year of the study period.

All comorbidities were identified using outpatient and inpatient care data. CCI for each patient was determined by summing up the points for all comorbidities for which a corresponding ICD-10-GM code was documented post-index. The treated FD patients were evaluated for FD-specific comorbidities during the total follow-up period. Different comorbidities specific to the involved organs were used as covariates (Table 1) for the logistic regression model to identify potential FD patients.

## Results

### Logistic regression model and assessing the probability of having FD

The model evaluated with covariates from approach one showed better diagnostic values: AIC (216.76 versus 220.50), SC (259.03 versus 258.54), and ROC (0.88 versus 0.88) than that with covariates from approach two (Table 1). Additionally, approach one showed prominent differences in organ involvement between the treated and control cohorts. The differences were checked by descriptive frequency tables. Based on these results, the model with covariates from approach one was selected. The list of covariates from approach one in the final model were used to identify potential FD patients.

The cut-point with the largest values for the Youden index (0.602174) and concordance probability method (0.64173) and the lowest value for closest to (0,1) (0.281343) was identified as 0.08 for each respective value. Patients with a cut-point probability >0.08 were identified as those with the highest probability of having FD and included in the potential FD cohort.

### Patient identification

In total, 814 patients were identified during the study period based on ICD-10-GM E75.2 diagnoses. The patient disposition in each cohort is described in Fig. 1. The treated FD cohort comprised 46 patients; control cohort included 460 subjects. A total of 284 patients with an E75.2 diagnosis but without FD-specific treatment and the probability of having FD higher than the cut-point were identified in the potential FD cohort using the logistic regression model; in addition, four patients with an additional diagnosis of “Glomerular disorders in other endocrine, nutritional and metabolic diseases” (ICD-GM-10 N08.4) were also included in the potential FD cohort (*N* = 288). The sensitivity and specificity for this cut-point were 80.4% and 79.8%, respectively.

### Patient demographics

The mean age of the treated FD patients (42.0 years) was higher than that of the potential FD patients (34.7 years) (Table 2). The proportion of younger patients (<20 years) was considerably lower in the treated FD cohort (6.5%) than in the potential FD cohort (38.2%). Further, there were more men in the treated cohort than in the potential FD cohort (Table 2). The mean age and gender distribution of the treated FD and control cohorts were comparable as the control cohort was a stratified sample based on the age and gender distribution of the treated FD cohort. Among the treated patients, agalsidase alfa (67.4%) was the most frequently prescribed initial treatment of choice, followed by agalsidase beta (23.9%) and migalastat (8.7%) during the total follow-up period.

**Table 2:** Demographic characteristics at the index date of study cohorts

Demographic characteristic	Potential FD cohort (*N* = 288)	Treated FD cohort (*N* = 46)	Control cohort* (*N* = 460)
**Age, years**			
Mean (SD)	34.7 (25.67)	42.0 (14.67)	43.3 (14.99)
Median (Q1–Q3)	29.0 (11.0–55.5)	46.0 (33.0–51.0)	46.0 (32.0–53.0)
**Age group**, **(*n *[%])**			
0–19	110 (38.2)	3 (6.5)	28 (6.1)
20–29	35 (12.2)	7 (15.2)	64 (13.9)
30–39	23 (8.0)	7 (15.2)	70 (15.2)
40–49	27 (9.4)	14 (30.4)	134 (29.1)
50+	93 (32.3)	15 (32.6)	164 (35.7)
**Gender**, **(*n *[%])**			
Male	135 (46.9)	27 (58.7)	270 (58.7)
Female	153 (53.1)	19 (41.3)	190 (41.3)
**Length of follow-up (quarters), years**			
Mean (SD)	13.3 (9.62)	21.2 (11.79)	23.9 (6.98)
Median (Q1–Q3)	12.0 (5.0–22.0)	27.0 (10.0–31.0)	28.0 (22.0–28.0)
**With four quarters of observation time**, **(*n *[%])**			
Pre-index	208 (72.2)	13 (28.3)	460 (100.0)
Post-index	243 (84.4)	39 (84.8)	460 (100.0)

*The control cohort was 10 times larger than the treated FD cohort

FD, Fabry disease; *n*, number of patients/individuals; Q, quartile; SD, standard deviation

### Incidence and prevalence of FD

The prevalence rate (per 100,000) of FD in the treated FD cohort increased from 0.60 in 2010 to 0.88 in 2017 (Table 3). The incidence rate (per 100,000) of FD in these patients was low and variable due to limited and even zero patient counts per year. The identification and incidence rates in the potential FD cohort in the German Sickness Fund Database were markedly higher than those in the treated FD cohort and increased from 2010 to 2017 (Table 3).

**Table 3:** Overall and annual incidence and identification/prevalence rates of FD in potential and treated FD cohorts

Year	Potential FD cohort (*N* = 288)		Treated FD cohort (*N* = 46)	
	Identification rate	Incidence rate	Prevalence rate	Incidence rate
**2010**	1.34	0.66	0.60	0.07
**2011**	1.29	0.70	0.69	0.07
**2012**	1.39	0.65	0.73	0.06
**2013**	1.40	0.60	0.68	0.00
**2014**	1.81	0.73	0.75	0.02
**2015**	2.01	0.79	0.72	0.02
**2016**	2.07	0.55	0.73	0.02
**2017**	2.38	0.78	0.88	0.09

Incidence and prevalence/identification rates are reported per 100,000 persons

FD, Fabry Disease

### Comorbidities

The comorbidities for treated and potential patients in each time-period were analyzed and compared with those of the control cohort to identify those with the highest prevalence differences between cohort A/B and cohort C. The FD-specific comorbidities in treated and potential cohorts during the total follow-up period were assessed using this algorithm (Table 4). While the mean CCI was comparable between the treated and potential FD patients, the median was lower in the treated patients (mean ± SD, 2.1 ± 2.36; median [range], 1.0 [0.0–4.0]) than in the potential FD patients (2.3 ± 2.29; 2.0 [0.0–3.0], respectively). Essential hypertension was the most common comorbidity in treated (46.2% and 38.5%) and potential (36.1% and 37.0%) FD cohorts pre-index and post-index, respectively. Over one-third of all patients in the treated FD cohort suffered from chronic kidney disease (35.9%) and dorsalgia (33.3%) post-index. In the potential FD cohort, more than one-third of patients reported disorders of refraction and accommodation pre-index (34.1%) and post-index (33.7%). The proportion of patients experiencing chronic ischemic heart disease and essential hypertension was higher in the treated FD cohort than that in the control cohort during pre-index. Chronic kidney disease, sequelae of cerebrovascular disease, and heart failure were more common among treated FD patients over the control cohort during post-index (Supplemental Table 1).

**Table 4:** FD-specific comorbidities in treated and potential FD cohorts during total follow-up period

Comorbidity*	Treated FD cohort (*N* = 46)	Potential FD cohort (*N* = 288)
**Peripheral nervous system, ** **(*n *[%])**		
Dizziness and giddiness	14 (30)	63 (22)
Conductive and sensorineural hearing loss	14 (30)	41 (14)
Tinnitus	12 (26)	32 (11)
Other and unspecified hearing loss	13 (28)	44 (15)
Disturbances of skin sensation	14 (30)	35 (12)
Other specified peripheral vascular diseases	7 (15)	5 (2)
Other chronic pain	8 (17)	51 (18)
**Gastrointestinal, ** **(*n *[%])**		
Nausea and vomiting	12 (26)	76 (26)
Gastroenteritis and colitis, unspecified and colitis, unspecified	11 (24)	73 (25)
Noninfective gastroenteritis and colitis, unspecified	6 (13)	40 (14)
Abdominal and pelvic pain	18 (39)	100 (35)
**Ophthalmological, ** **(*n *[%])**		
Hereditary cornea dystrophies	5 (11)	5 (2)
**Renal, ** **(*n *[%])**		
Chronic kidney disease	30 (65)	76 (26)
Unspecified kidney failure	8 (17)	50 (17)
Proteinuria	6 (13)	17 (6)
**Cardiac, ** **(*n *[%])**		
Heart failure	17 (37)	50 (17)
Cardiomyopathy	12 (26)	22 (8)
Cardiomegaly	14 (30)	6 (2)
Malaise and fatigue	7 (15)	38 (13)
Atrial fibrillation and atrial flutter	7 (15)	33 (11)
Hypertensive heart disease with (congestive) heart failure	6 (13)	14 (5)
Cardiac arrhythmia, unspecified	6 (13)	25 (9)
**Cerebrovascular, ** **(*n *[%])**		
Cerebral transitory ischemia and related syndromes	8 (17)	26 (9)
Transient cerebral ischemia, unspecified	6 (13)	19 (7)
Stroke	15 (33)	41 (14)
Stroke, not referred to as bleeding or infarction	10 (22)	43 (15)
**Neuropsychological, ** **(*n *[%])**		
Other anxiety disorders	6 (13)	41 (14)
**Pulmonary, ** **(*n *[%])**		
Dyspnea	5 (11)	37 (13)
Breathing disorders	8 (17)	56 (19)
Cough	8 (17)	45 (16)
**Lymphatic, ** **(*n *[%])**		
Edema, not elsewhere classified	10 (22)	33 (11)

Incidence and prevalence/identification rates are reported per 100,000 persons. *Comorbidities with *n* ≥5 for both cohorts were included, based on a list of pre-selected comorbidities common in patients with FD. At least one occurrence of comorbidity in the total follow-up period was considered for inclusion. All comorbidities specified are according to the ICD-10-GM code. The BKK German Sickness Fund Database consists of ICD-10-GM codes, which do not provide exact and specific diagnosis; instead, the codes provide diagnostic categories into which specific and precise diagnoses are added even though the codes are not always exactly appropriate

BKK, *BetriebsKrankenKassen*; FD, Fabry Disease; ICD-10-GM, International Statistical Classification of Diseases and Related Health Problems, 10^th^ revision, German Modification; *n*, number of patients/individuals

Comorbidities with the highest difference in the potential FD cohort compared to those in the control cohort were disorders of refraction and accommodation and other strabismus during pre-index, and acute upper respiratory infections of multiple and unspecified sites and epilepsy during post-index. Nausea and vomiting were more common in the potential FD cohort than in the control cohort during pre-index and post-index (Supplemental Table 1).

### Sensitivity analysis

A sensitivity analysis of the potential FD cohort was performed to investigate possible changes in the results when applying a more restrictive rule to identify these patients. The sensitivity analysis cohort included patients selected in the potential FD cohort, i.e., those with a probability of having FD higher than the 0.08 cut-point determined via logistic regression and additionally with at least two E75.2 diagnoses during the observation period. This was comparable with the treated FD cohort, wherein at least two E75.2 diagnoses were documented for almost the entire cohort. Of 288 patients in the potential FD cohort, 139 had at least two ICD-10-GM E75.2 diagnoses in the total follow-up period and were identified as the sensitivity analysis cohort.

In Germany, diseases (especially chronic ones) are documented not only when initially diagnosed but also when treated. As FD is a severe chronic disease, it is likely that it is documented more than once during a period of several years. A second or third diagnosis need not be related to an actual new “diagnosis” by the same/another physician but could also be just the documentation of an existing disease (i.e., FD).

Patient demographics were comparable between the potential FD and sensitivity analysis cohorts (Supplemental Table 2). As expected, the identification and incidence rates of FD in the sensitivity analysis cohort (2.67 and 1.87, respectively) were lower than those in the potential FD cohort (5.35 and 4.35, respectively) due to more restrictive patient selection rules. There were many similarities with respect to comorbidities of interest typical of FD between the potential FD and sensitivity analysis cohorts (Supplemental Table 2). In the sensitivity analysis cohort, the CCI (mean ± SD, 2.6 ± 2.3; median [range], 2.0 [1.0–3.0]) was comparable with that of the potential FD cohort (2.3 ± 2.3 and 2.0 [0.0–3.0], respectively).

## Discussion

We developed a logistic regression model to characterize potential FD patients, identified based on the ICD-10-GM coding system, using a sickness fund database from Germany. We optimized the specificity and sensitivity of the model to determine a likely threshold for identifying potential FD patients. The robustness of the results for the potential FD cohort was further validated by a sensitivity analysis that used stricter inclusion criteria for identifying potential FD patients, resulting in comparable comorbidities between the potential FD cohort and the sensitivity analysis cohort.

Overall, the treated FD patients were older than the potential FD patients. Although male patients with classic FD manifest disease-specific symptoms during childhood, it often takes up to 15 years before FD is diagnosed. The applied algorithm identified a considerable number of potential FD patients. Half of the potential FD patients (145/288 patients) were younger (below the age of 30 years), perhaps because renal or cardiac FD symptoms were not yet clinically overt and remained untreated for FD. In contrast, only 6.5% of the treated FD patients (3/46 patients) were below the age of 20 years and another seven (15.2%) patients were aged between 20 and 29 years. These findings support the relevance and clinical value of the algorithm, which may facilitate timely diagnosis and early treatment of patients with FD.

While it is not surprising that the potential FD cohort has more younger patients than that of the FD treated cohort [10, 22], the data also suggest that treatment is more commonly initiated among male than female patients [23]. There were more female patients in the potential FD cohort than in the treated cohort. This might be expected since FD-specific signs and symptoms are often more prominent and severe in classic male patients than in female patients. Due to the random inactivation of one of the two X chromosomes and depending on the type of mutation and the level of residual α-Gal activity, women can have a variable disease course, frequently exhibit a slow disease progression, and become symptomatic later in life [24–26]. Hence, the diagnosis might be missed or delayed more often in female than in male patients with FD. The higher proportion of younger and of female patients in the potential FD cohort than that in the treated FD cohort suggests that delayed treatment initiation may be determined by typically less severe symptoms of FD in the younger and female patient groups.

While the applied algorithm did not grade symptom severity, it used the same criteria to identify younger and older or male and female potential FD patients. The higher proportion of young and female patients in the potential FD cohort than in the treated FD cohort might not only be attributed to symptom manifestation at a later age in general but also to lower symptom severity in female patients with FD in particular. Instead, other criteria may delay treatment initiation, such as reluctance to expose young patients to a lifelong, costly treatment, or misperception of female patients with FD as less affected compared to male patients who need no continuous expensive treatment [22, 23].

The yearly prevalence/identification rates of FD increased over time in treated and potential FD cohorts. Similarly, the incidence rate of FD in the potential cohort increased over time. However, the incidence rate of FD in the treated cohort was variable due to only single or even no patients with FD in respective years. The identification and incidence rates of the potential FD patients in this study were higher than those in published literature [5, 27] possibly owing to underreporting of FD [3, 28]. In addition, the heterogeneity of disease spectrum ranging from classical to non-classical phenotypes, large intersex variability, and difference in disease severity [3, 8, 9] contribute to missed diagnoses leading to underreporting. Besides, in Germany, a unique ICD code specific to FD is lacking. The ICD-10-GM E75.2 is not unique for FD but is an amalgamation of all lysosomal storage diseases, including sphingolipidoses, FD, Gaucher disease, Krabbe, Niemann–Pick, Farber syndrome, Metachromatic leukodystrophy, and Sulfatase deficiency. Thus, our estimates could be overreported due to potential misclassification of the patients. The incidence and prevalence rates of FD in the sensitivity analysis cohort with stricter inclusion criteria were lower than those in the potential FD cohort but were higher than those in the treated FD cohort.

FD is associated with a wide range of comorbidities involving different organs, particularly the heart, kidneys, or eyes [3]. We observed a similar comorbidity profile in the treated FD cohort. Chronic ischemic heart disease and essential hypertension were the most frequently reported comorbidities during pre-index. In contrast, chronic kidney disease or heart failure were more frequently reported during post-index. These cardiac and renal complications might have contributed to FD-specific treatment initiation. Disorders of refraction and accommodation, other strabismus, nausea and vomiting, and abdominal and pelvic pain were more frequently reported comorbidities in the potential FD cohort than in the control cohort.

We acknowledge the limitations associated with the use of an insurance claims database. It is unlikely that only one diagnosis is reported in the database for a severe disease like FD. This might be due to administrative mistakes with claims while noting the ICD-10 code or milder disease severity. Cases wherein a patient stops follow-up with the clinician who made the diagnosis and does not inform the new doctor about it, can lead to misreporting in the database [3, 28, 29]. However, this is mainly applicable for potential FD patients as all treated patients except one (with only a single diagnosis in the last quarter of 2017, i.e., the last year of the observation period) reported at least two diagnoses of FD. Hence, no further diagnosis could be made. Furthermore, the number of treated FD patients used to build the model was limited and included old patients (only 6.5% patients were aged <20 years). Thus, there is some uncertainty about the model in detecting patients in the younger age group without FD-specific comorbidities. Although the study design allowed the identification of patients’ comorbidities before and after the first visible diagnosis, medical history or diagnoses outside of the pre-defined time before index date (up to 1 year) were not captured, thereby underreporting the number/frequency of certain comorbidities, especially acute diseases or symptoms. The datasets used/built for the study analysis are a part of the BKK German claims Database and are anonymized and not directly available due to national data protection laws. Thus, further follow-up on the 288 potential FD patients was not possible within the scope of this study.

There are some limitations of the logistic regression model. First, the logistic regression model was built with covariates defined by diagnoses identified via ICD-10 GM codes from an insurance claims database. As with any claims databases, these data are subject to data coding limitations and data entry errors. Second, the BKK database consists of ICD-10-GM codes that do not provide exact and specific diagnoses but provide diagnostic “categories” into which specific and precise diagnoses are added, resulting in certain instances of misclassification. Third, information on results of examinations, such as blood or genetic testing, were unavailable. Therefore, the selection of potential FD patient could not be confirmed by the results of these examinations. Lastly, validation of the model was not within the planned scope of this study but should be considered in the future for further applications of this algorithm.

This algorithm was developed and applied within the context of the present study design. Application of this algorithm elsewhere would require further development, adjustment, and evaluation, which might further increase its sensitivity/specificity. In the future, it will be interesting to analyze such data by splitting the records into training and testing cohorts; however, such an approach could not be achieved in this study because of limited sample size. Moreover, the applicability of this algorithm outside Germany needs to be evaluated as it is dependent on data availability from different claims databases and country-level differences in claims reporting. However, given the available data (properly collected in our health care systems) used for the development of this algorithm, this model can be adapted for use.

Overall, given the challenges associated with definitive and timely FD diagnosis, this is one of the first regression models that can identify and characterize potential FD patients using clinical signs of the disease. The model outputs were validated by applying strict criteria for patient identification, which adds to the robustness of this model. Future applications of this model may include evaluation of individuals of a database not diagnosed with FD or other sphingolipidosis and identify those with high likelihood as they show characteristics similar to those of treated FD patients according to their comorbidity profile. Additional applications of this model are being considered to better diagnose and manage FD patients, including the incorporation of medical results that are not captured within claims data.

## Conclusions

The model developed in this study served as a tool to identify potential FD patients using BKK German claims database. The results from sensitivity analysis highlighted that most comorbidities were comparable between the potential FD and sensitivity analysis cohorts and showed minor differences in demographics. This further suggests that the identified potential FD cohort can be considered robustly evaluated. Patients with FD experience long odysseys in their diagnostic pathway from symptom presentation to diagnosis and eventual treatment. This model provides useful insights to physicians on the identification of potential FD patients using available clinical information. The symptoms in the potential FD patients may be less severe than those among the treated FD patients, suggesting discrepancy in the disease burden that accounts for missing treatment among potential FD patients.

### Electronic supplementary material

Below is the link to the electronic supplementary material.

Supplementary Material 1

## Data Availability

The datasets used/built for the analysis are not directly available and are subject to national data protection laws.

## Abbreviations

AIC: Akaike information criterion
CCI: Charlson Comorbidity Index
FD: Fabry disease
ICD-10-GM: International Statistical Classification of Diseases and Related Health Problems, 10^th^ revision, German Modification
ROC: receiver operating characteristic
SD: Standard Deviation
