# Supplementary material for: Unveiling the untreated: development of a database algorithm to identify potential Fabry disease patients in Germany
Source: Orphanet J Rare Dis. 2024 Jul 9;19:259. doi: 10.1186/s13023-024-03258-y (PMC11234697; doi:10.1186/s13023-024-03258-y)
Supplement: Supplementary file 1 — Supplementary Material 1 [file 13023_2024_3258_MOESM1_ESM.docx]

# Supplementary Materials

**
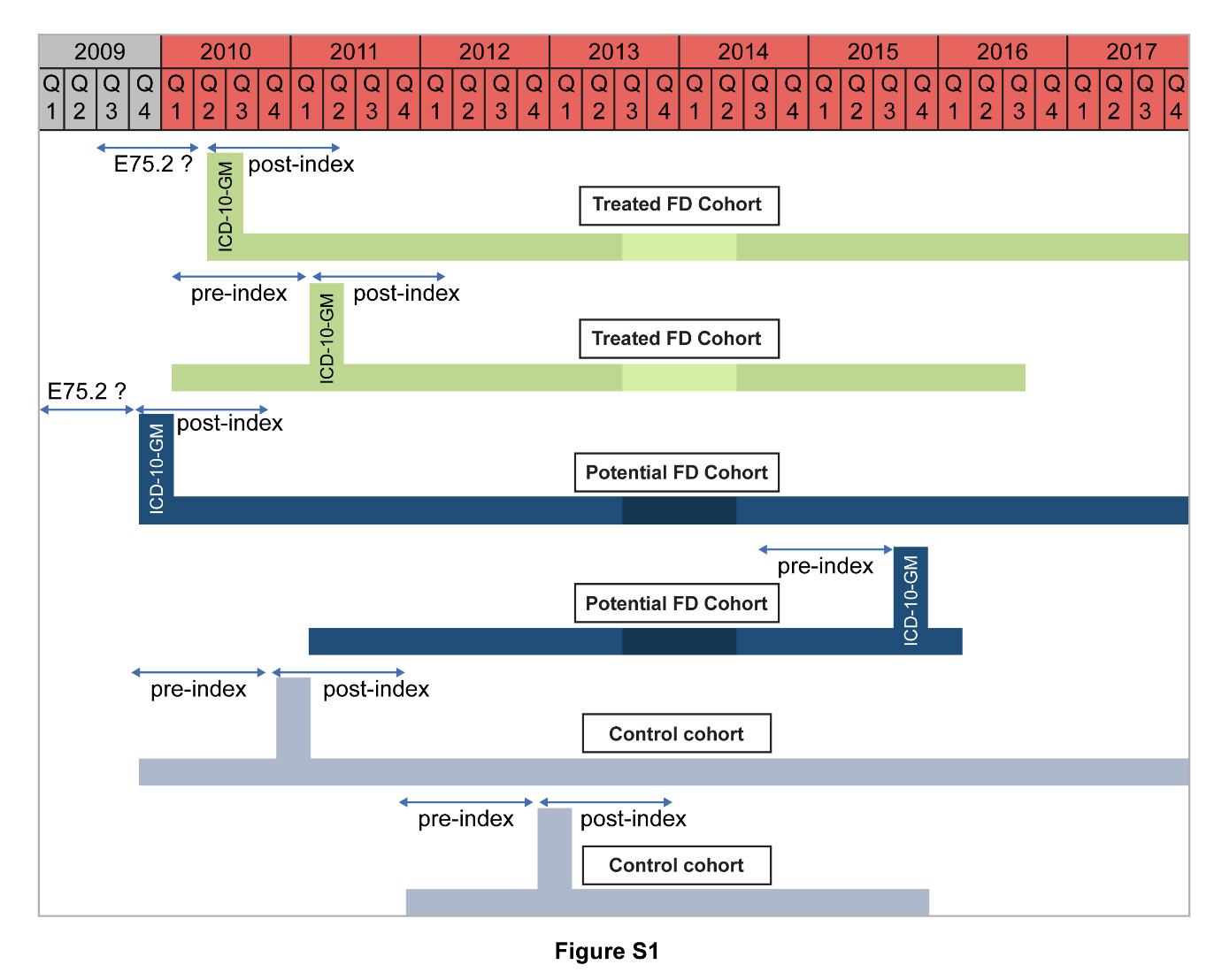
Supplemental Fig. 1.** Examples for the study period, time before and after index for treated FD cohort (green), potential FD cohort (blue), and control cohort C (grey).

FD, Fabry disease; ICD-10-GM, German version of the ICD-10 diagnosis code.

**Supplemental Table 1** Top-ten comorbidities with highest differences in prevalence between the treated/potential FD cohort and control cohort in outpatient and inpatient care – pre- and post-indexes.

| **Comorbidity/diagnosis** | **Treated/ potential cohort**  ***n* (%)*** | **Control cohort  *n* (%)** |
| --- | --- | --- |
| **Treated FD patients, before index** | | |
| Chronic ischemic heart disease | 5 (39%) | 53 (12%) |
| Essential (primary) hypertension | 6 (46%) | 163 (35%) |
| **Potential FD patients, before index** | | |
| Disorders of refraction and accommodation | 71 (34%) | 93 (20%) |
| Other strabismus | 37 (18%) | 24 (5%) |
| Enduring personality changes, not attributable to brain damage and disease | 24 (12%) | 0 (0%) |
| General examination and investigation of persons without complaint and reported diagnosis | 55 (26%) | 70 (15%) |
| Infantile cerebral palsy^#^ | 24 (12%) | 2 (0) |
| Specific developmental disorder of motor function | 23 (11%) | 2 (0%) |
| Mixed specific developmental disorders^†^ | 23 (11%) | 3 (11%) |
| Unspecified disorder of psychological development | 22 (11%) | 2 (0) |
| Nausea and vomiting | 29 (14%) | 20 (4%) |
| Visual disturbances | 38 (18%) | 40 (9%) |
| **Treated FD patients, after index** | |  |
| Chronic kidney disease | 14 (36%) | 26 (6%) |
| Sequelae of cerebrovascular disease | 6 (15%) | 6 (1%) |
| Heart failure | 7 (18%) | 23 (5%) |
| Cardiomyopathy | 6 (15%) | 13 (33%) |
| Unspecified kidney failure | 6 (15%) | 15 (3%) |
| Other disorders of cornea | 5 (13%) | 4 (1%) |
| Other peripheral vascular diseases | 6 (15%) | 16 (4%) |
| Conductive and sensorineural hearing loss | 6 (15%) | 19 (4%) |
| Stroke, not referred to as bleeding or infarction | 5 (13%) | 10 (2%) |
| Nonrheumatic mitral valve disorders | 5 (13%) | 11 (2%) |
| **Potential FD patients, after index** | |  |
| Acute upper respiratory infections of multiple and unspecified sites | 76 (31%) | 75 (16%) |
| Epilepsy | 40 (17%) | 15 (3%) |
| Disorders of refraction and accommodation | 82 (34%) | 97 (21%) |
| Mixed specific developmental disorders | 30 (12%) | 2 (0%) |
| Nausea and vomiting | 35 (14%) | 12 (3%) |
| Other disorders of urinary system | 49 (20%) | 39 (9%) |
| Unspecified disorder of psychological development | 29 (12%) | 2 (0) |
| Infantile cerebral palsy^#^ | 28 (12%) | 1 (0%) |
| Other functional intestinal disorders | 31 (13%) | 7 (3%) |
| Unspecified urinary incontinence | 32 (13%) | 10 (2%) |

Not all comorbidities listed in Supplemental Table 1 were necessarily conditions related to FD.

^#^Nomenclature as used in the German version of ICD-10: [ICD-10-GM-2023: G80.- Infantile Zerebralparese - icd-code.de](https://www.icd-code.de/suche/icd/code/G80.-.html?sp=Sg80) (Source: BfArM - ICD-10-GM Version 2023 (dimdi.de)

^†^Nomenclature as used in the German version of ICD-10: ICD-10-GM-2023: F83 Kombinierte umschriebene Entwicklungsstörungen - icd-code.de (Source: BfArM - ICD-10-GM Version 2023 (dimdi.de)

*Comorbidities with *n* ≥ 5 for both cohorts included.

FD, Fabry Disease; *n*, number of patients

**Supplemental Table 2** Baseline demographics and comorbidities of interest in the potential FD cohort and the sensitivity analysis cohort.

| **Demographic characteristics** | **Potential FD cohort**  ***N* = 288** | **Sensitivity analysis cohort**  ***N* = 139** |
| --- | --- | --- |
| **Age (years)** | | |
| Mean (SD) | 34.7 (25.67) | 35.1 (26.4) |
| Median (Q1–Q3) | 29.0 (11.0–55.5) | 32.0 (9.0–58.0) |
| **Age group (years), *n* (%)** | | |
| 0–19 | 110 (38.2%) | 54 (38.8%) |
| 20–29 | 35 (12.2%) | 14 (10.1%) |
| 30–39 | 23 (8.0%) | 11 (7.9%) |
| 40–49 | 27 (9.4%) | 13 (9.4%) |
| 50+ | 93 (32.3%) | 47 (33.8%) |
| **Gender, *n* (%)** | | |
| Male | 135 (46.9%) | 59 (42.4%) |
| Female | 153 (53.1%) | 80 (57.6%) |
| **Comorbidity, *n* (%)** | | |
| Gastrointestinal | 186 (65.0%) | 92 (66.0%) |
| Ophthalmological | 77 (27.0%) | 39 (28.0%) |
| Renal | 103 (36.0%) | 52 (37.0%) |
| Cerebrovascular | 88 (31.0%) | 48 (35.0%) |
| Neuropsychological | 117 (41.0%) | 53 (38.0%) |
| Pulmonary | 103 (36.0%) | 50 (36.0%) |

FD, Fabry Disease
